# Early Events of the Reaction Elicited by CSF-470 Melanoma Vaccine Plus Adjuvants: An *In Vitro* Analysis of Immune Recruitment and Cytokine Release

**DOI:** 10.3389/fimmu.2017.01342

**Published:** 2017-10-23

**Authors:** María B. Pampena, María M. Barrio, Estefanía P. Juliá, Paula A. Blanco, Erika M. von Euw, José Mordoh, Estrella Mariel Levy

**Affiliations:** ^1^Centro de Investigaciones Oncológicas-Fundación Cáncer, Buenos Aires, Argentina; ^2^UCLA JCCC-Translational Oncology Research Labs, Los Angeles, CA, United States; ^3^Instituto Médico Especializado Alexander Fleming, Buenos Aires, Argentina; ^4^Fundación Instituto Leloir, IIBBA-CONICET, Buenos Aires, Argentina

**Keywords:** immunotherapy, melanoma vaccine, innate immunity, leukocyte recruitment, pro-inflammatory cytokines, bacillus Calmette–Guerin, adjuvants

## Abstract

In a previous work, we showed that CSF-470 vaccine plus bacillus Calmette–Guerin (BCG) and granulocyte macrophage colony-stimulating factor (GM-CSF) as adjuvants resulted in a significant benefit in the distant metastasis-free survival when comparing vaccinated *vs*. IFN-α2b-treated high-risk cutaneous melanoma patients in a Phase II study. Immune monitoring demonstrated an increase in anti-tumor innate and adaptive immunities of vaccinated patients, with a striking increase in IFN-γ secreting lymphocytes specific for melanoma antigens (Ags). In an effort to dissect the first steps of the immune response elicited by CSF-470 vaccine plus adjuvants, we evaluated, in an *in vitro* model, leukocyte migration, cytokine production, and monocyte phagocytosis of vaccine cells. Our results demonstrate that leukocytes recruitment, mostly from the innate immune system, is an early event after CSF-470 vaccine plus BCG plus GM-CSF interaction with immune cells, possibly explained by the high expression of CCL2/MCP-1 and other chemokines by vaccine cells. Early release of TNF-α and IL-1β pro-inflammatory cytokines and efficient tumor Ags phagocytosis by monocytes take place and would probably create a favorable context for Ag processing and presentation. Although the presence of the vaccine cells hampered cytokines production stimulated by BCG in a mechanism partially mediated by TGF-β and IL-10, still significant levels of TNF-α and IL-1β could be detected. Thus, BCG was required to induce local inflammation in the presence of CSF-470 vaccine cells.

## Introduction

Cutaneous melanoma (CM) originates from melanocytes, and it is an immunogenic tumor, as suggested by the presence of regression areas observed in primary tumors and by the correlation between “brisk” lymphocytic infiltrates and longer survival (1). In recent years, immunotherapy became a promising option and blockade of immune checkpoints with monoclonal antibodies (MAbs) has shown relevant clinical results in metastatic disease, further implying the importance of lymphocytes to control tumor progression (2–4). On the other hand, vaccination approaches to foster the immune system reactivity have had limited success in CM patients, probably because most studies were performed treating patients with advanced disease using a few single antigens (Ags) as immunogens (5–7). In view of the multiplicity of mutated and non-mutated Ags present in CM (8, 9), our approach to vaccination has been: (i) to use irradiated whole tumor cells as a source of multiple tumor Ags, providing the immune system with the opportunity to process them without *a priori* selection by the investigator (10–12), and (ii) to overcome the possible immune tolerance toward tumor cells by adding bacillus Calmette–Guerin (BCG) and granulocyte macrophage colony-stimulating factor (GM-CSF) as adjuvants. We have previously determined, in Phase I clinical trial in which 20 CM patients were treated with fixed doses of an allogeneic vaccine plus BCG and variable doses of GM-CSF that 400 µg of GM-CSF per vaccination (divided into four daily injections) was optimal (10). Strikingly, 64% of the stage III CM patients from that study remain relapse-free after a median follow-up >192 months. A closely similar vaccine (CSF-470) was assayed in Phase II clinical study, in which 31 CM patients stage IIB, IIC, and III were randomized to receive CSF-470 vaccine plus BCG and GM-CSF versus medium-dose IFN-α2b (13). CSF-470 is a mixture of four CM cell lines to which whole exome sequencing and mRNA expression analysis were performed; the cell lines are heterogeneous for HLA class I haplotype and for expression of melanocytic differentiation Ags (11, 13). In that study, with a mean and maximum follow-up of 39.4 and 83 months, respectively, a significant benefit in the distant metastasis-free survival of vaccinated patients was observed (*p* = 0.022) as compared to IFN-α2b (medium dose). Immune monitoring demonstrated an increase in anti-tumor innate and adaptive immunity of vaccinated but not in IFN-α2b-treated patients with a striking increase in IFN-γ-secreting lymphocytes specific for melanoma Ags (13). Therefore, a TH_1_ immune response was induced by this vaccination system. Despite numerous clinical studies demonstrating BCG and GM-CSF abilities to activate immune responses (14–17), we could not find any reports in which the *in vitro* interactions between tumor cells and these adjuvants were studied. To dissect the first steps of such interactions we evaluated, in an *in vitro* model, leukocyte migration, cytokine production and monocyte phagocytosis. Our results demonstrate that leukocyte recruitment, mostly from the innate immune system, is an early event after CSF-470 vaccine plus BCG plus GM-CSF interaction with immune cells. In fact, CCL2/MCP-1 chemokine released by CSF-470 vaccine cells could account, at least partially, for monocytes and lymphocytes attraction. Early release of TNF-α and IL-1β pro-inflammatory cytokines and efficient tumor Ags phagocytosis by monocytes also occurs and would favor subsequent Ag processing and presentation. Finally, although the presence of the vaccine cells partially inhibited cytokines production stimulated by BCG, still significant levels of TNF-α and IL-1β could be detected. Thus, BCG was required to induce local inflammation in the presence of CSF-470 vaccine cells.

## Materials and Methods

### CSF-470 Vaccine Cells

The CSF-470 vaccine consists of lethally gamma-irradiated cells (apoptotic/necrotic) derived from four CM cell lines established in-house from human metastatic melanoma tumors. The cell lines MEL-XY1, MEL-XY2, MEL-XY3 and MEL-XX4 were grown in a GMP core facility at the Centro de Investigaciones Oncológicas—FUCA and were cultured as previously described (11). Vaccine preparation was formerly described (13). In all *in vitro* experiments irradiated CSF-470 cells were used (CSF-470 vaccine).

### RNASeq

RNASeq was performed for each viable cell line with the sequencing platform Illumina Hiseq 4000, with more than 20 M high-quality single-end reads per sample (BGI Americas). Quality control of reads was performed with FASTX-Toolkit^1^ and FastQC.^2^ Reads were aligned to the latest human Hg38 reference genome using the STAR spliced read aligner (18). Fragment counts were derived using HTSeq package (19). Differentially expressed genes were identified by a ranking based on adjusted *p*-values ≤0.005 and a false discovery rate≤0.1 using the R/Bioconductor package edgeR (20). RNASeq data from the melanoma cell lines were uploaded to the European Nucleotide Archive (EMBL-EBI). The corresponding accession numbers are as follows: JM1: ERS1949828 (SAMEA104324850); JM2: ERS1949829 (SAMEA104324851); JM3: ERS1949830 (SAMEA104324852); and JM4: ERS1949831 (SAMEA104324853). Samples correspond to JM1: MEL-XY1 cell line; JM2: MEL-XY2 cell line; JM3: MEL-XY3 cell line; and JM4: MEL-XX4 cell line.

### CCL2/MCP-1 and IL-10 Production by CSF-470

To determine and quantify chemokines or cytokines produced by CSF-470 vaccine or by the composing cell lines, 5 × 10^5^ cells were placed in 24-well plates in 1 mL of RPMI 1640 medium (Thermo Fisher Scientific, USA). After 6, 24, and 48 h incubation, cells were harvested and centrifuged at 1,500 rpm for 5 min. Supernatants were collected and stored at −80°C until further analysis. Chemokine and cytokine levels were measured with ELISA kits (BD Biosciences, USA) following the manufacturer’s instructions. Color intensity was measured at 450 nm in a BioRad plate reader. The standard curves and concentrations were calculated using GraphPad Prism 5.0 (USA).

### Obtention of Leukocytes, Peripheral Blood Mononuclear Cells (PBMCs), and Monocytes

Peripheral blood was obtained from healthy donors (HDs) at the “Instituto Médico Alexander Fleming.” This study was carried out in accordance with the recommendations of “Comité de Etica del Instituto Médico Especializado Alexander Fleming.” Blood samples from HD were obtained according to the Instituto Médico Alexander Fleming guidelines, at the Hemotherapy Service. Leukocytes were obtained after lysis of red blood cells with pH 7.3 ammonium-chloride buffer solution during 15 min, washed with phosphate-buffered saline (PBS), incubated for another 10 min, followed by a second wash with PBS. PBMCs were purified using a Ficoll density gradient (GE Healthcare, UK), and monocytes were isolated using an anti-CD14^+^ cell kit (Miltenyi, Germany) following the manufacturer’s specifications.

### *In Vitro* Migration Assay

Leukocyte chemotaxis was determined using HTS Transwell-24 units with 0.5 µm pore polycarbonate membranes and 6.5 mm inserts (Costar, Corning, NY, USA). A total of 600 µL of plain RPMI 1640 medium with the eventual addition of 5 × 10^5^ CSF-470 vaccine cells, 10 µg/mL GM-CSF (Laboratorio Pablo Cassará, Argentina), or 10,000 CFUs of BCG (Pasteur strain, Instituto Malbrán, Argentina) were added to the bottom wells. The top wells were loaded with 5 × 10^5^ leukocytes or 1 × 10^6^ PBMCs in 100 µL RPMI and incubated for 6 h at 37°C in a 5% CO_2_ incubator (Figure S1A in Supplementary Material). After removing the inserts, the cells in the bottom plate were harvested, washed, and incubated with antibodies for FACS analysis.

### FACS Analysis of Migrating Cells

Leukocytes or PBMCs were incubated with PerCP anti-human CD45 MAb (clone 2D1) to distinguish between immune and CSF-470 vaccine cells. Figure S1B in Supplementary Material shows the gating strategy to analyze polymorphonuclear cells, lymphocytes, and monocytes. PE anti-CD3 (clone SK7) and APC anti-CD56 (clone B159) MAbs were used to select T and natural killer (NK) lymphocytes, respectively. From the T-cell population (CD3^+^), we also analyzed CD8^+^ cells with FITC anti-CD8 (clone RPA-T8). To characterize classical and nonclassical monocytes, we used FITC anti-CD16 (clone 3G8) and PE anti-CD14 (clone M5E2). Isotype-matched irrelevant MAbs were used as negative controls. All MAbs were from BD Biosciences. Data acquisition was performed using FACSCanto II cytometer (BD Biosciences, USA), collecting total CD45^+^ events in the bottom well. Data analysis was performed with FlowJo software 10.0.7 (Tree Star Inc., USA).

### Phagocytosis Assay

CSF-470 vaccine cells were stained with PKH67 (staining protocol as stated on the datasheet, Sigma, USA) and cultured with CD14^+^ purified monocytes with or without BCG and GM-CSF, for 4, 24, or 48 h at 37 or 4°C, followed by 15 min room temperature incubation with PE-CD14 (clone M5E2). PKH67 incorporation by CD14^+^ cells was assessed on FACSCanto II Flow Cytometer (BD Biosciences). Data were analyzed using FlowJo software. Percentage of monocytes that has phagocytosed labeled material from CSF-470 vaccine was calculated as the proportion of PKH67^+^ cells within the CD14^+^ population.

### PBMCs and Monocytes Stimulation

A total of 5 × 10^5^ PBMCs or purified monocytes were cultured in 1 mL RPMI 1640 medium supplemented with 10% heat-inactivated fetal calf serum, 2 mM glutamine, 100 U/mL penicillin, and 100 µg/mL streptomycin, alone or in the presence of 5 × 10^5^ CSF-470 vaccine cells with or without adjuvants (160,000 CFUs of BCG plus 10 µg/mL GM-CSF). When indicated, 10 µg/mL anti IL-10 and anti TGF-β inhibitory MAbs were added (anti IL-10 clone 23738, anti TGF-β clone 1D11 or control isotypes mouse IgG2b clone 20116 and mouse IgG1 clone 11711; R&D Systems, USA). After 6, 24, and 48 h incubation the medium was harvested and centrifuged at 1,500 rpm for 5 min. Supernatants were collected and stored at −80°C until further analysis. TNF-α, IL-1β, IL-10, and IL-12 cytokines were measured in coculture supernatants using ELISA kits (BD Biosciences) as described.

### Statistical Analysis

Statistical tests for migration assays and coculture experiments for cytokines measurements were done with Infostat 2011 software (21). Data were analyzed according to a randomized block design with repeated measures (time) using analysis of variance, considering time, treatments and their interactions as fixed factors, and HD as a random factor (blocks); α = 0.05. All models were tested for homoscedasticity and normality of residuals by visual assessment of plots. When homoscedasticity was not accomplished, models were fitted by the addition of the VarIdent, VarExp, or VarPower variance structure to the random part of the model (22). The best variance structure used in the fitted models was determined by comparison of Akaike’s and Bayesian’s Information Criteria. For monocytes and lymphocytes proportion comparison in migration assays, paired *T*-test was used (GraphPad Prism 5.0). A *p* value <0.05 was considered to be statistically significant.

## Results

### CSF-470 Plus Adjuvants Attracted Leukocytes and PBMCs

**Figure 1:**
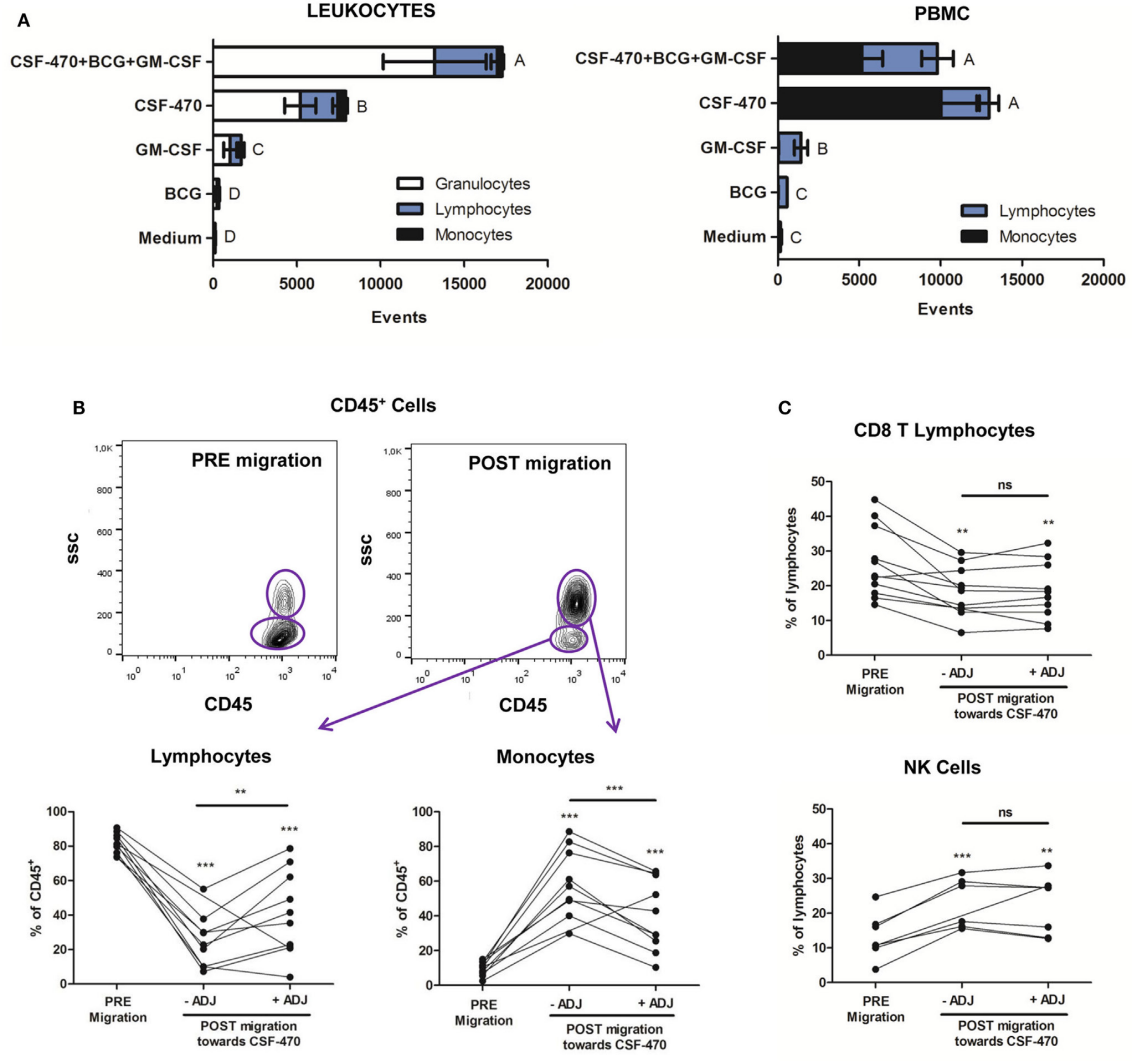
Leukocyte and mononuclear cell migration toward CSF-470 vaccine. **(A)** Left panel: Leukocyte migration showed as event counts toward each component: adjuvants bacillus Calmette–Guerin and granulocyte macrophage colony-stimulating factor (ADJ), CSF-470 vaccine or vaccine plus adjuvants, *n* = 11. **(A)** Right panel: Mononuclear cell migration is shown as event counts toward each component: individual adjuvants, CSF-470 vaccine or vaccine plus adjuvants, *n* = 10. Bars with different letters are statistically different (*p* < 0.05). **(B)** Frequency of monocytes and lymphocytes in PRE- and POST-migrated CD45^+^ population toward the vaccine with or without adjuvants (paired *T*-test; *p < 0.01, **p < 0.001; ***p < 0.0001). **(C)** Frequency of CD3^+^CD8^+^ T cells and natural killer cells in PRE- and POST-migrated lymphocyte population toward CSF-470 vaccine with or without adjuvants (paired *T*-test; *p < 0.01, **p < 0.001; ***p < 0.0001), *n* = 8.

Since CSF-470 vaccine is injected intradermally with GM-CSF and BCG, we aimed to establish an *in vitro* coculture system to analyze the possible interaction between vaccine components and immune cells that may occur *in vivo*, with the limitation that no extracellular matrix, blood or lymphatic vessels were present. We first analyzed leukocyte migration. After 6 h incubation, the combination of CSF-470 plus adjuvants promoted stronger leukocyte migration than the individual components (Figure 1A, left). In decreasing order, CSF-470 and GM-CSF alone significantly attracted leukocytes, whereas BCG was not an attractor. We also analyzed which leukocyte sub populations were attracted. Granulocytes were the most attracted cells, followed by lymphocytes and monocytes, thus maintaining the same proportion as in peripheral blood (Figure 1A, left).

In order to explore the migration of potentially phagocytic immune cells, and due to the small percentage of monocytes in the total leukocyte sample, we decided to purify PBMC that led to an increase in the initial proportion of this cell subpopulation. When the migration assay was performed with PBMC instead of total leukocytes, we found that migration was mainly promoted by CSF-470 vaccine, while the presence of adjuvants did not modify such attraction (Figure 1A, right). After migration to CSF-470, monocytes were enriched and became the prevalent population (9.8 ± 3.9% PRE migration *vs*. 59.37 ± 19.82% POST migration; *p* < 0.001; Figure 1B, lower right panel). Lymphocytes were also attracted but reducing their proportion after migration (81.6 ± 5.95% PRE migration *vs*. 24.79 ± 15.43% POST migration toward CSF-470; *p* < 0.001; Figure 1B, lower left panel). When adjuvants were added, monocytes were also highly attracted (9.8 ± 3.9% PRE migration *vs*. 40.2 ± 20.43% POST migration toward CSF-470 plus adjuvants; *p* < 0.001; Figure 1B, lower right panel). Furthermore, migrated lymphocytes to CSF-470 vaccine or CSF-470 vaccine plus adjuvants showed an increase in the proportion of NK cells as compared to the pre-migration lymphocyte population, in detriment of CD3^+^CD8^+^ T cells (Figure 1C).

Phenotype assessment showed that monocyte CD14, CCR7, and MHC class II expressions were unchanged among treatments. Also, activation markers CD25 and CD69 remained low and constant in T lymphocytes and NK cells (data not shown). To summarize, cells from the innate immune system such as monocytes and NK cells appear to be preferentially attracted by CSF-470 vaccine.

### Classical Monocytes Are Attracted by CSF-470 Vaccine and Phagocytose-Irradiated Cells

**Figure 2:**
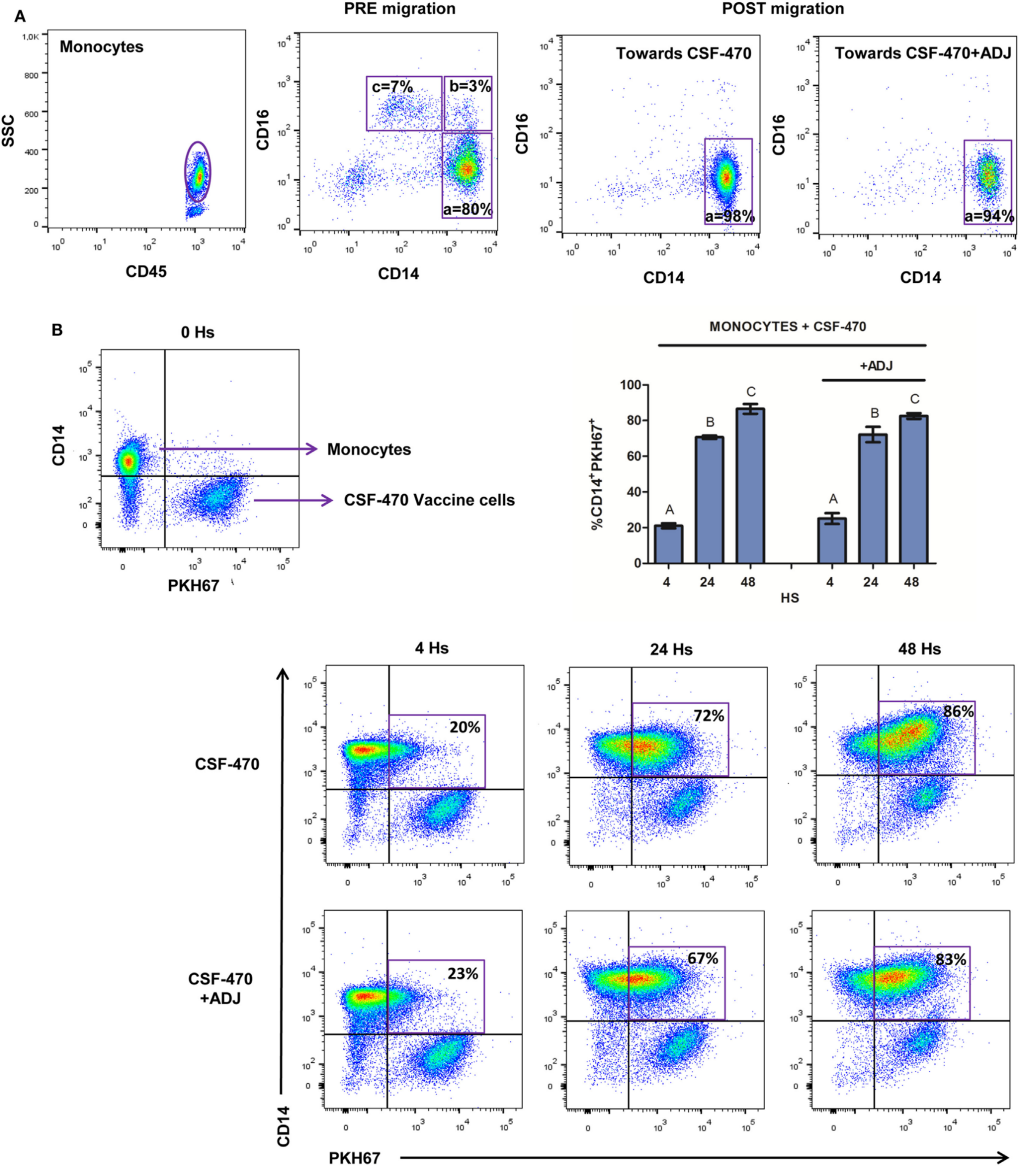
Classical monocytes migration and phagocytosis of irradiated cells. **(A)** Proportion of classical (a = CD14^++^CD16^−^), intermediate (b = CD14^++^CD16^+^), and nonclassical monocytes (c = CD14^+^CD16^++^) PRE and POST migrations toward CSF-470 vaccine, with or without adjuvants bacillus Calmette–Guerin and granulocyte macrophage colony-stimulating factor (ADJ). One representative experiment is shown. **(B)** Phagocytosis of CSF-470 vaccine cells at 4, 24, and 48 h. Selected gates represent the percentage of monocytes that have phagocytose-labeled material from CSF-470 vaccine. Bars represent the percentage of monocytes from healthy donor (*n* = 3) that uptake irradiated CSF-470 cells (double staining CD14^+^ PKH67^+^) at 4, 24, and 48 h. Bars with different letter are statistically different (*p* < 0.05). To illustrate, representative dot plots from one experiment are depicted.

After PBMC migration, cells were harvested from the lower well and monocytes were characterized by CD14 and CD16 surface marker expression. CSF-470 vaccine, with or without adjuvants, preferentially attracted CD14^++^CD16^−^ classical phagocytic monocytes (Figure 2A). To test the phagocytic capacity of monocytes, purified CD14^+^ cells were incubated with PKH67-labeled CSF-470 vaccine cells, with or without adjuvants. After 4 h coculture, 21.0 ± 1.4% of monocytes had captured cell-labeled material; this uptake increased to 70.7 ± 0.9 and 86.5 ± 2.8% at 24 and 48 h, respectively (Figure 2B). Phagocytosis was similar when adjuvants were present (4 h: 25.1 ± 3.0%; 24 h: 72.2 ± 4.3%; 48 h: 82.5 ± 1.6%).

### CSF-470 Cells Produce High Amount of Chemokines

**Figure 3:**
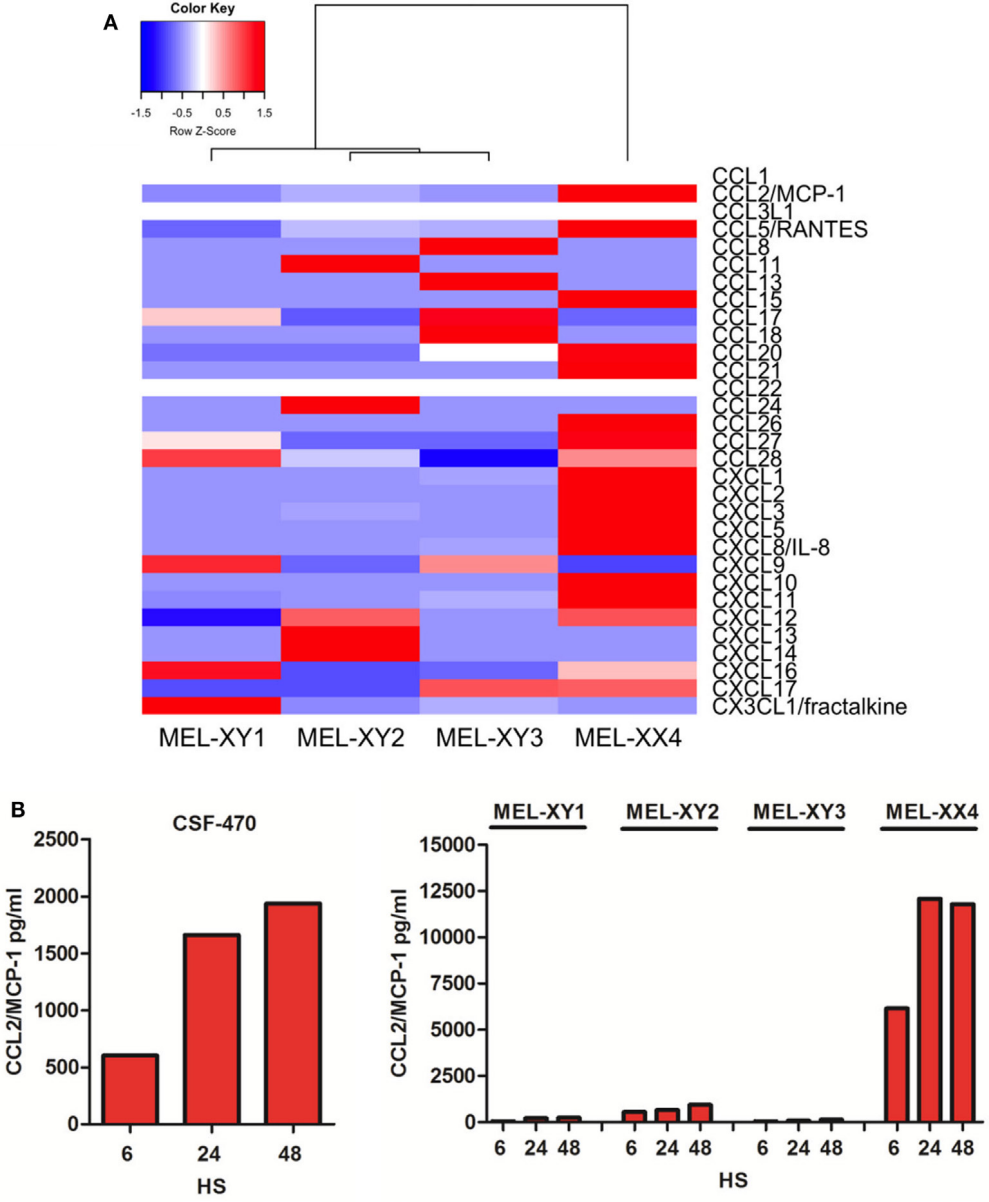
Chemokine expression profile of cutaneous melanoma cell lines components of CSF-470 vaccine. **(A)** Chemokines expression by non-irradiated MEL-XY1, MEL-XY2, MEL-XY3, and MEL-XX4 cells by RNAseq. Transcript abundance for each cell line is shown as fragments per kilobase of exon per million fragments mapped. **(B)** CCL2/MCP-1 protein expression at 6, 24, and 48 h supernatants from CSF-470 vaccine cells and each irradiated cell line.

Monocytes and NK cells migrate to inflammatory sites in response to several chemokines (23, 24). RNAseq analysis allowed us to determine the expression profile of the four viable cells that compose the CSF-470 vaccine as shown in Figure 3A. MEL-XX4 cell line expresses high amounts of neutrophils chemoattractants CXCL1, CXCL2, and CXCL3. MEL-XX4, and to a lesser extent MEL-XY2, also express high amounts of CCL2/MCP-1. To validate these results, we measured CCL2/MCP-1 protein secretion by irradiated CSF-470 cells after 6, 24, and 48 h of culture. It is worth mentioning that CCL2/MCP-1 mRNA expression by non-irradiated cells (RNAseq) and protein secretion to the conditioned medium from irradiated cells completely matched (Figure 3B). We evaluated PBMC chemoattraction to each cell line component of the vaccine separately. When tested individually, we observed that MEL-XX4 alone attracted more cells than the other three cell lines, although this tendency was not statistically significant (data not shown). Thus, additional chemoattractant factors, other than CCL2/MCP-1 may be responsible for monocyte and lymphocyte attraction.

### BCG Addition to CSF-470 Vaccine Triggers Monocyte Production of Pro-inflammatory Cytokines

**Figure 4:**
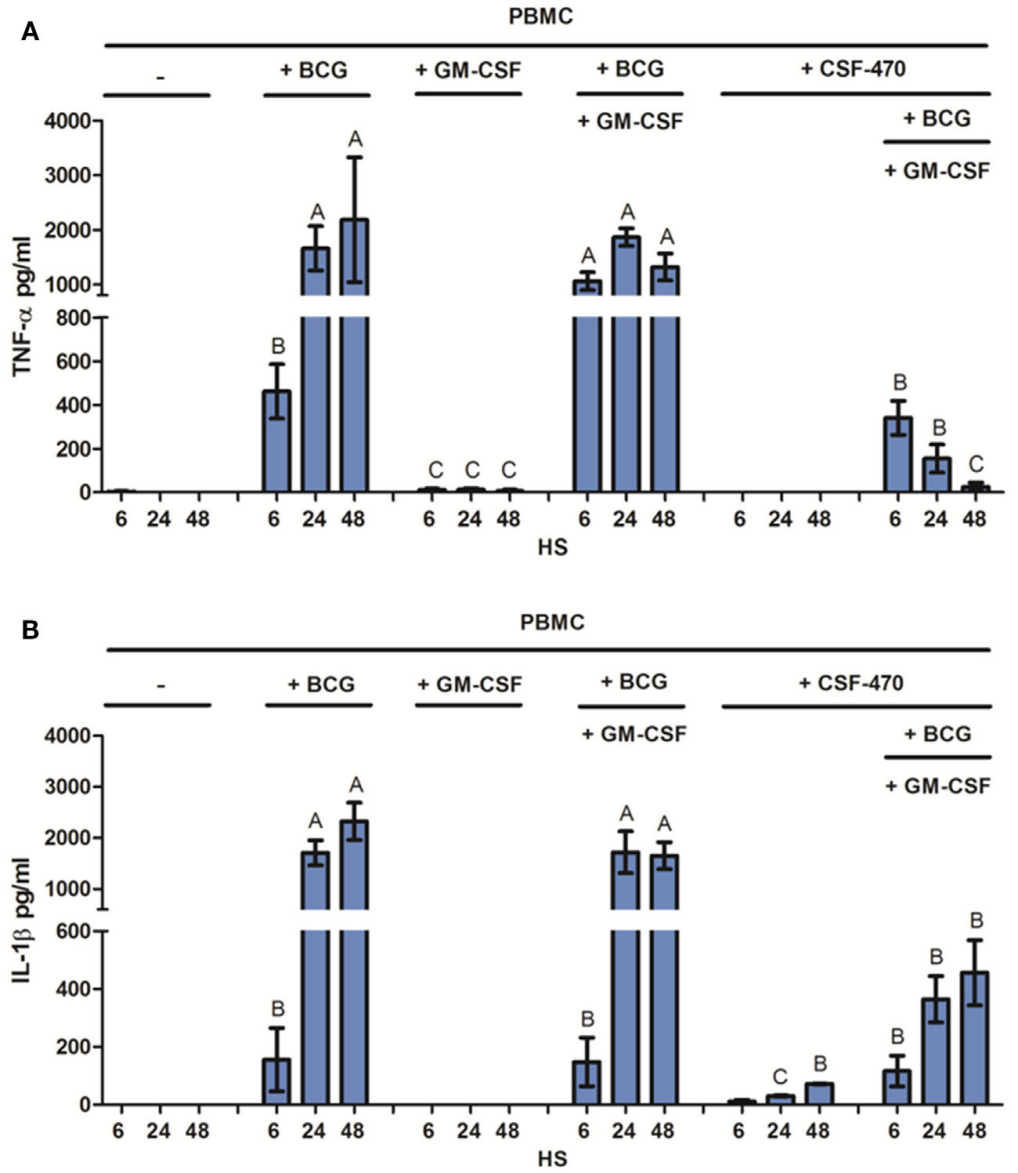
Pro-inflammatory cytokines released by peripheral blood mononuclear cell (PBMC) in CSF-470 cocultures with or without adjuvants, after 6, 24, and 48 h. **(A)** TNF-α released by PBMC, *n* = 6. **(B)** IL-1β released by PBMC, *n* = 3. Bars with different letters are statistically different (*p* < 0.05).

Monocyte activation is associated with the release of pro-inflammatory cytokines, mainly TNF-α and IL-1β (25). Thus, we measured the production of these cytokines in cocultures of PBMC and adjuvants BCG and GM-CSF and/or CSF-470. CSF-470 by itself was unable to induce secretion of either of these cytokines by PBMC. The low levels of IL-1β observed in PBMC–CSF-470 cocultures without adjuvants correspond to the production of this cytokine by CSF-470 vaccine cells (Figure S2 in Supplementary Material). Instead, BCG, but not GM-CSF, induced a high release of TNF-α and IL-1β, leading to cytokine accumulation for 48 h (Figure 4). At the first 6 h, GM-CSF increased TNF-α release induced by BCG, but in the following time, it did not contribute to its production. On the other hand, GM-CSF had no impact on IL-1β release. The presence of the vaccine cells partially inhibited cytokines production stimulated by BCG, but still significant levels of TNF-α and IL-1β could be detected. Thus, BCG was required to induce local inflammation in the presence of CSF-470 vaccine cells (Figure 4). When cocultures were performed using purified CD14^+^ monocytes or peripheral blood lymphocytes from PBMCs, monocytes were the leading producers of TNF-α and IL-1β at least during the first 48 h (data not shown).

In order to quantify the BCG induction of TNF-α release by PBMC and its inhibition by CSF-470 cells, we performed concentration curves. Results are depicted in Figure S3 in Supplementary Material. TNF-α release showed a BCG dose-dependent pattern, characterized by the highest release at 24 h with 500,000 CFUs of BCG, the highest amount tested. Opposite to that, increasing amounts of CSF-470 cells decreased TNF-α release in a dose-dependent way. IL-12 could not be detected in these cocultures (data not shown).

### CSF-470 Produces High Amounts of Immune Suppressive Molecules

Bacillus Calmette–Guerin is directly recognized by TLR-2 and TLR-4 in monocytes and actively stimulates TNF-α and IL-1β production (26). In our setting, BCG alone or with GM-CSF efficiently induced both inflammatory cytokines; however, the presence of CSF-470 vaccine significantly reduced pro-inflammatory cytokines secretion. To investigate the mechanisms that could explain that phenomenon, we evaluated by RNAseq the expression of several immune suppressive factors commonly produced by tumor cells.

**Figure 5:**
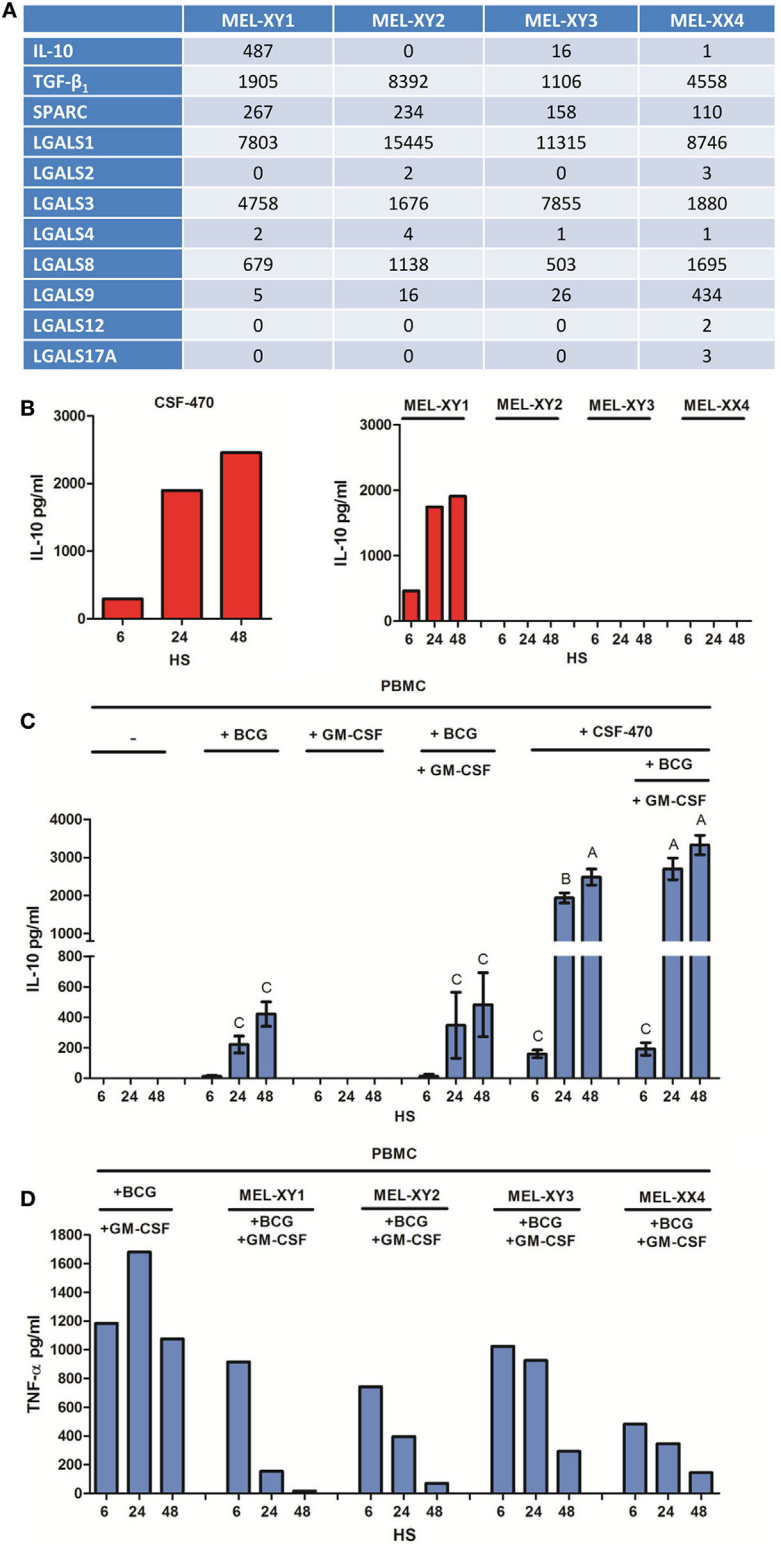
Anti-inflammatory cytokine production by CSF-470 vaccine. **(A)** IL-10, TGF-β, SPARC, and galectin expressions by RNAseq from MEL-XY1, MEL-XY2, MEL-XY3, and MEL-XX4 cell lines. **(B)** IL-10 detected in conditioned media of CSF-470 or each irradiated cell lines after 6, 24, and 48 h. **(C)** IL-10 detected in supernatants of peripheral blood mononuclear cell (PBMC) cocultures with CSF-470 with or without adjuvants. Bars with different letters are statistically different (*p* < 0.05). **(D)** TNF-α release by PBMC exposed to each irradiated cell line plus adjuvants bacillus Calmette–Guerin and granulocyte macrophage colony-stimulating factor.

Analysis of the four cell lines comprising CSF-470 vaccine showed abundant expression of SPARC, TGF-β, IL-10, and galectins 1, 3, 8, and 9 mRNAs (Figure 5A). Of note, MEL-XY1 cell line was the only IL-10 producer as demonstrated both at the mRNA and protein levels (Figures 5A,B). Furthermore, the high levels of IL-10 cytokine detected in supernatants of PBMC cocultures with CSF-470 plus adjuvants came mostly from CSF-470 vaccine cells (Figure 5C). However, cocultures of PBMC with the individual cell lines comprising the CSF-470 vaccine showed that inhibition of TNF-α release mediated by MEL-XY1 was similar to the inhibitory effect mediated by non-expressing IL-10 cell lines (Figure 5D). Therefore, other immunosuppressive factors are probably contributing to the hampered TNF-α release by PBMC exposed to the vaccine.

### IL-10 and TGF-β Are Responsible for CSF-470 Vaccine Immunosuppression

**Figure 6:**
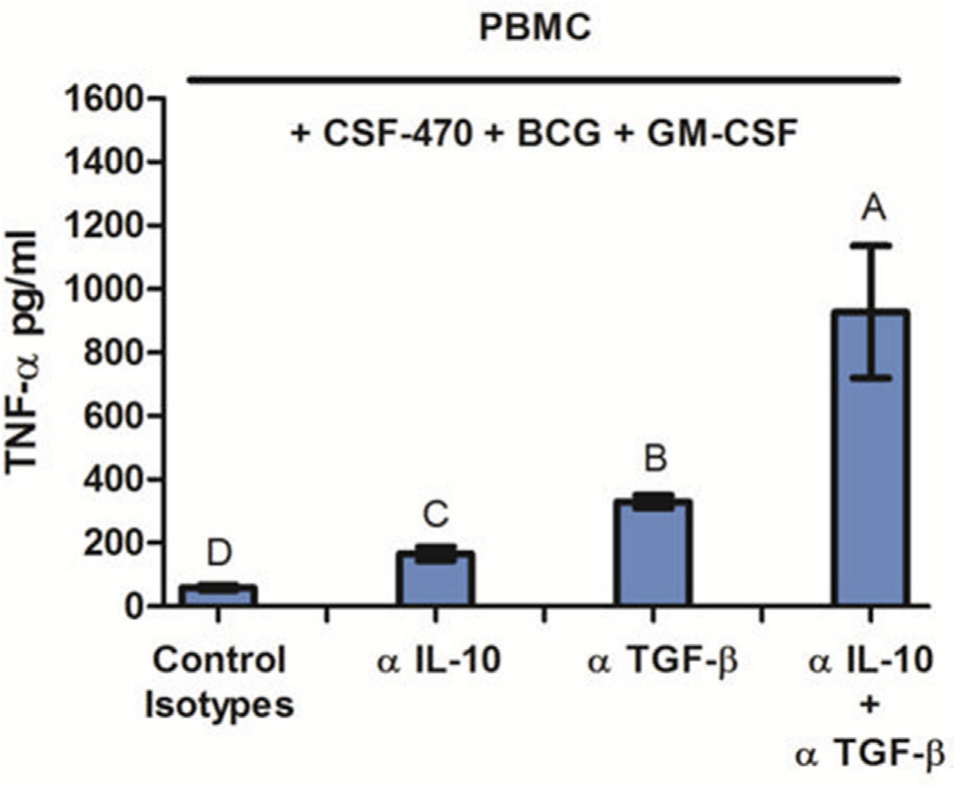
TNF-α release by peripheral blood mononuclear cell coculture with CSF-470 plus adjuvants at 24 h in the presence of blocking antibodies against IL-10 and/or TGF-β. Bars with different letters are statistically different (*p* < 0.05), *n* = 3.

Finally, in order to demonstrate the immune suppressive role of IL-10 and TGF-β produced by CSF-470 vaccine, we measured TNF-α released by PBMC cocultures with CSF-470 plus adjuvants, in the presence of blocking MAbs against IL-10 and/or TGF-β. Figure 6 shows that blocking IL-10 and TGF-β increased TNF-α secretion by PBMC after 24 h coculture with CSF-470 vaccine plus adjuvants, suggesting that these cytokines strongly hampered TNF-α secretion induced by BCG addition.

## Discussion

The current state of the art proposes that for a cancer vaccine to be useful in the development of an immunological response, Ag-presenting cells must capture Ags and process them, activate and mature, traffic to the lymph nodes (LNs), and prime naive T cells. Alternatively, Ags could be captured directly at the LN level. Immunological activation would translate into a clinical response by trafficking of T cells into tumors, activation of T-cell effector functions after engagement of tumor Ag, and consequent lysis of tumor cells.

In the previously described Phase II clinical trial, CSF-470 vaccine plus BCG and GM-CSF as adjuvants was administrated at frequent intervals for a total of 13 vaccinations over a two-year period. Besides challenging the immune system with a broad repertoire of CM Ags (27) and non-identical HLAs, the treatment strategy included the use of BCG in every vaccination to induce local inflammation, polarize immune cells toward a TH_1_ response and activate NK cell cytotoxicity and memory-like response (28, 29). Moreover, GM-CSF was injected with every vaccination during 4 days presumably contributing to differentiate monocytes into dendritic cells (DCs) as shown *in vitro* (30). In that clinical trial, immune monitoring demonstrated significant increases in peripheral T cells reactive against melanoma Ags, peripheral NK cells frequency, and a specific antibody response. However, the events that take place at the vaccination site remain speculative since the *in situ* reaction starts within hours, and it lasts for weeks. Another hindrance for that analysis is that sequential biopsies of the injection site are difficult to obtain; besides, the inflammatory changes do vary with subsequent vaccinations. In the present work, using an *in vitro* model similar to that used to evaluate migration to a DC vaccine (31), we analyzed the first steps of the interaction among CSF-470, BCG, GM-CSF, and leukocytes from HD. We established that leukocytes migration was promoted within hours, with CSF-470 being the highest attractor. Innate immune cells were mostly attracted, and among them, neutrophils, probably because CXCL1 and CXCL2 chemokines, which mediate neutrophils recruitment (32, 33), were highly expressed by MEL-XX4 cells. When we focused on PBMC, monocyte was the main migrating mononuclear cells. Since it has been reported that monocytes role on vaccination depends on their subtype (34–36), we determined that the attracted monocytes mainly belonged to the CD14^++^CD16^−^ classic phagocytic type. This subtype represents approximately 85% of the CD14^+^ population and expresses CD62L, CCR2, CLEC4D, CLEC5A, IL13Ra1, CXCR1, and CXCR2 (25). CCL2/MCP-1 regulates the migration and infiltration of monocytes, memory T lymphocytes, and NK cells (37). Considering that high mRNA and protein levels of CCL2/MCP-1, the ligand of the CCR2, were found in CSF-470, this chemokine may partially contribute to monocytes and NK migration, at least within the first 48 h; however, it should be confirmed by blocking CCL2/MCP-1 in the migration assay.

It has been reported by others (38) that CCL2/MCP-1 exogenously administered or produced locally at inflammatory sites, could drain into regional LNs, where it binds to high endothelial venules and triggers circulating monocyte binding and diapedeses. In vaccinated patients, assuming that not every CSF-470 Ags are phagocytosed *in situ* and that in experimental models only about 2% of Ag-loaded DC reach the draining LN (39, 40), such blood-derived monocytes could phagocytose tumor Ags directly drained into LNs and further trigger naive lymphocytes education. However, our *in vitro* assay is quite simple and does not allow us to understand further events that may take place at longer times of interaction. It is probable that monocytes have multiple roles in this setting, besides cytokine production, as described by Italiani et al. (41). Either tissue monocytes or blood monocytes recruited in response to inflammatory stimuli have been shown to give rise to the inflammatory monocyte-derived macrophages, while some of them do not differentiate into macrophages and remain monocyte-like cells. They can take up Ag and migrate to the draining LNs (tissue monocytes); these are the Ag-uptaking and -presenting cells of the tissue. Although we have evidenced monocyte phagocytosis of tumor irradiated cells in this setting, we have not evaluated T-cell priming. Given that up to 48 h of interaction we did not observe upregulation of CCR7 (data not shown), these CD14^+^ monocytes might still not be able to differentiate to Ag-presenting cells and migrate to LNs to allow canonical T-cell priming. However, events that would take longer to occur and/or be induced by monocyte interaction with other components of the inoculation site such as other immune cells, stroma, and vascular cells, could not be evaluated in our *in vitro* assays. It is hard to ascertain, which is the origin of the Ag-presenting cells in the dermis after vaccination. The vaccination nodules are located well below the epidermis (42), so it would not appear probable that epidermal residing Langerhans cells are directly involved in the first Ag-presenting process. However, their role after repeated vaccinations may not be discarded, since it has been demonstrated that under inflammatory conditions, Langerhans cells may induce a shift from skin resident, T regulatory memory lymphocytes to effector memory lymphocytes (43).

Even in the case of BCG used as an inflammatory stimulant for noninvasive bladder carcinoma, where research has been extensively pursued, the role of monocyte/macrophages in BCG response has been suggested but not confirmed *in vivo*, evidencing the complexity of appropriate models to study these multiple interactions. However, an increase in IFN-γ release by lymphocytes of patients treated with an antitumoral vaccine was observed when BCG was used as an adjuvant (44).

Bacillus Calmette–Guerin dramatically induced TNF-α and IL-1β release by monocytes at high nM levels, leading to cytokines accumulation peaking at 24 h for TNF-α and at least 48 h for IL-1β. As to the mechanism by which BCG acts on monocytes, it is well known that it interacts with multiple receptors such as TLR-2, TLR-4, and NLRs, stimulating innate immunity and leading to specific adaptive immunity (45). Among the benefits of released TNF-α on vaccination, it may be quoted that attained levels may induce production of endothelial leukocyte adhesion molecule 1 by endothelial cells (46) and therefore enhance leukocyte entry into the vaccination site. Other positive effects of TNF-α and IL-1β release stems from the fact that prior work from our laboratory demonstrated that both cytokines are necessary to restore the mixed lymphocyte reaction ability of DCs after phagocytosis of tumor irradiated cells (47). On the other hand, if not counterbalanced, high TNF-α release by monocyte activation could be detrimental for the long range activation of the immune system. Although its role in fibrotic diseases is still controversial, the TNF-α pro-inflammatory properties in the initial phases of fibrosis development have been deeply characterized in experimental models (48). Therefore, an enhanced granulomatous reaction would encapsulate the vaccination site and consequently, hinder the access of Ag-presenting cells to the antigenic focus and the exit of particulate material to draining LNs. CSF-470 by itself did not induce cytokines release by monocytes, and it diminished about 20-fold the BCG activity on such release.

Vaccine cell lines expressed IL-10, galectins 1, 3, and 8, SPARC, and TGF-β, which induce immune suppression in a variety of ways (49–54). In fact, IL-10, released only by MEL-XY1 cell line, is considered the most important cytokine preventing inflammation-mediated tissue damage (49). Furthermore, the immunosuppressive effect of CSF-470 on TNF-α release by monocytes could be partially reversed by blocking TGF-β and IL-10, suggesting an important role for these cytokines, at least during the first 48 h.

In the analyses here performed, GM-CSF appeared to play a minor role, and we suggest this is so because this cytokine would act at later stages of vaccination. On one hand, it would enhance endothelial proliferation and subsequent PBMC efflux to the injection site. In fact, a substantial erythema appeared at the injection site in vaccinated patients when GM-CSF was added, which lasted for 2–4 days and then subsided (13). The analysis of this possible effect of GM-CSF could not be performed since no endothelial cells were added to our system. On the other hand, GM-CSF would drive monocyte differentiation into DCs, a late effect that was not analyzed in our system.

Thus, as a final result, a balance between pro-inflammatory and anti-inflammatory chemokines and cytokines might be established at the vaccination site. It could be assumed that, if given alone, CSF-470 would act as a sink for PBMC, attracting them by expressing a variety of chemokines and, through immunosuppressive factors, blocking the release of inflammatory cytokines by PBMC. BCG enters this equation by partially reversing such inhibition, and it is, therefore, an essential player. It is hard to link the early *in vitro* events determined in this paper with the immune stimulation obtained in patients after several vaccinations (13); nevertheless, they offer a glimpse of the events taking place after the first vaccination. Establishing experimental systems that allow analyzing cell interactions in a longer time frame is highly desirable.

## Ethics Statement

This study was carried out in accordance with the recommendations of “Comite de Etica del Instituto Medico Especializado Alexander Fleming.” Blood samples from healthy donors were obtained according to the Instituto Medico Alexander Fleming guidelines, at the Hemotherapy Service.

## Author Contributions

MP, JM, and EL collected and assembled the data, analyzed and interpreted the data, and wrote the manuscript. MB analyzed and interpreted the data and wrote the manuscript. EJ analyzed and interpreted the data and wrote the manuscript. PB collected and assembled the data. EE collected and assembled the data and analyzed and interpreted the data.

## Conflict of Interest Statement

The authors declare that the research was conducted in the absence of any commercial or financial relationships that could be construed as a potential conflict of interest.

## Footnotes

^1^http://hannonlab.cshl.edu/fastx_toolkit/.

^2^http://www.bioinformatics.babraham.ac.uk/projects/fastqc/.

## Abbreviations

MAb, monoclonal antibodies; Ags, antigens; BCG, bacillus Calmette–Guerin; CM, cutaneous melanoma; DCs, dendritic cells; HD, healthy donor, PBMCs, Peripheral blood mononuclear cells; PBS, phosphate saline buffer; GM-CSF, recombinant human granulocyte macrophage colony-stimulating factor; LN, lymph node.
