# Supplementary figures and images for: Early Events of the Reaction Elicited by CSF-470 Melanoma Vaccine Plus Adjuvants: An In Vitro Analysis of Immune Recruitment and Cytokine Release
Source: Front Immunol. 2017 Oct 23;8:1342. doi: 10.3389/fimmu.2017.01342 (PMC5660290; doi:10.3389/fimmu.2017.01342)

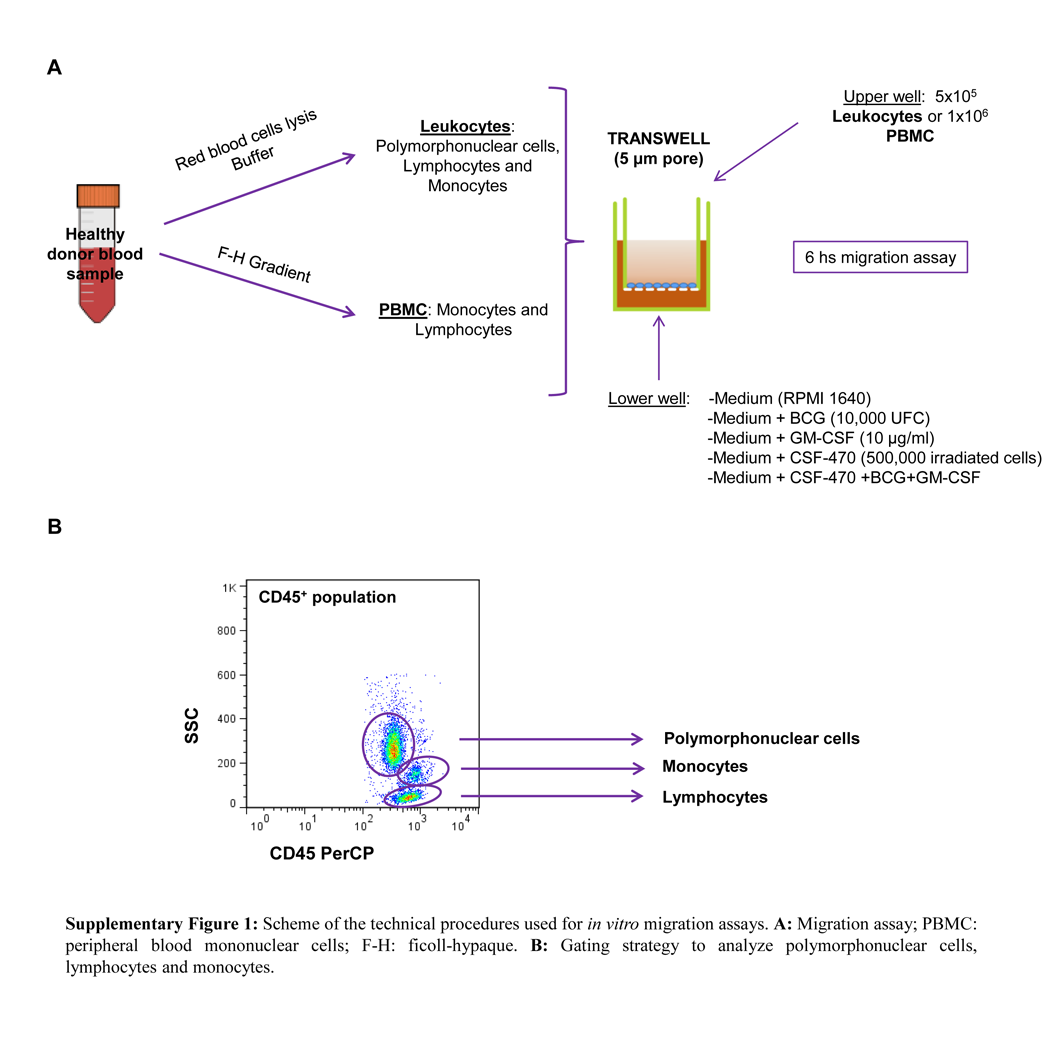

Supplement: Supplementary file 1 [file Image_1.tif]

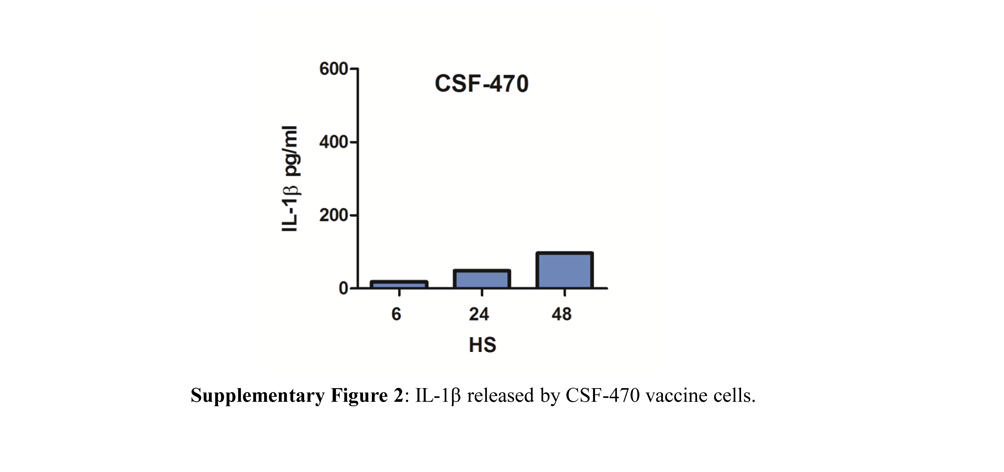

Supplement: Supplementary file 2 [file Image_2.TIF]

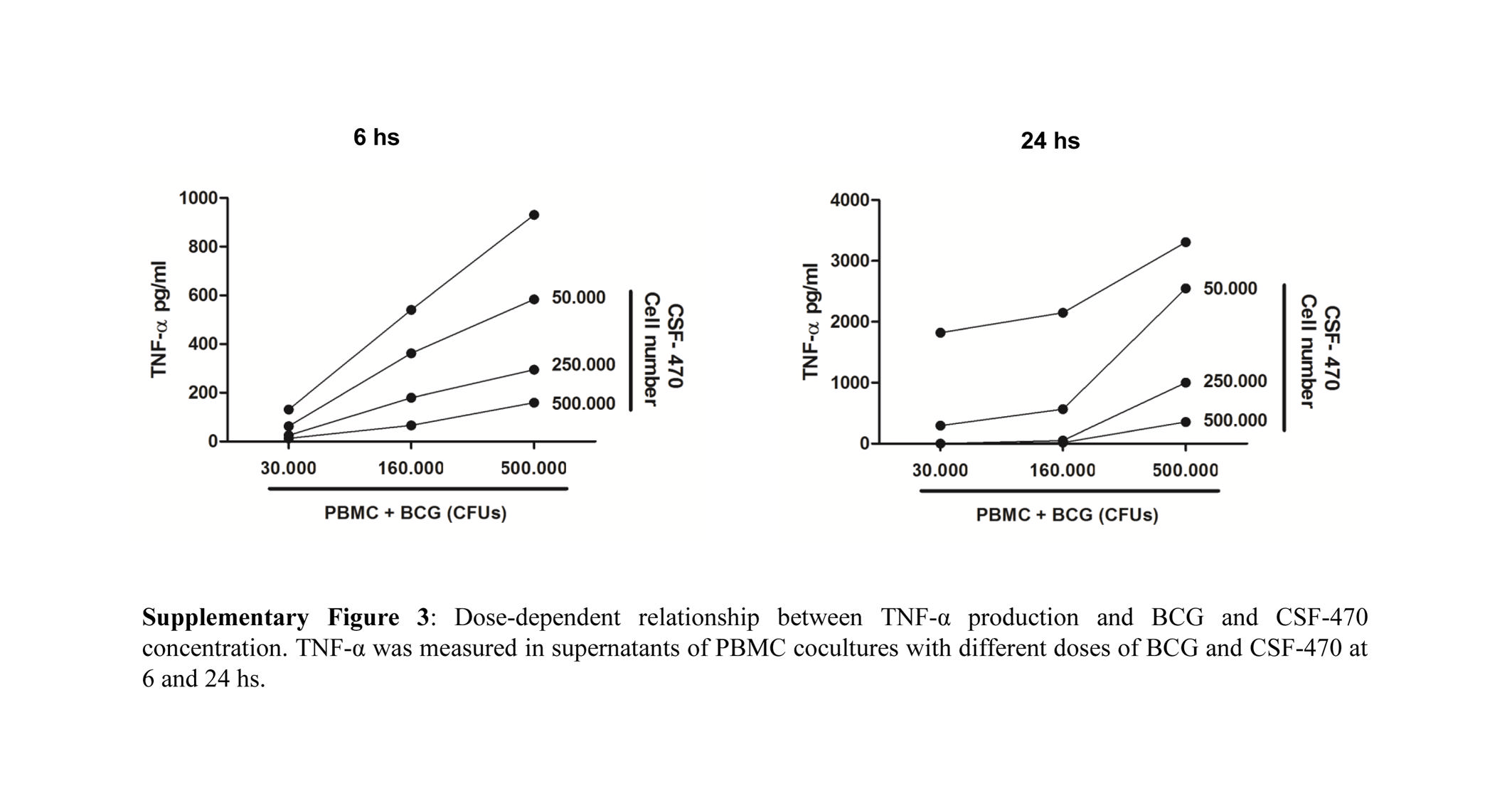

Supplement: Supplementary file 3 [file Image_3.TIF]
